# The Prevalence of the Celiac Disease Among Urban Bedouin Population in Israel

**DOI:** 10.4021/gr464e

**Published:** 2012-07-20

**Authors:** Rudoy Inna, Korobeinikov Andrew, Shalev Hanna, Volkov Ilia

**Affiliations:** a Department of Family Medicine, Sial Research Center for Family Medicine and Primary Care, Faculty of Health Sciences, Ben-Gurion University of the Negev, Beer-Sheva, Israel; b Clalit Health Services, Southern District, Beer-Sheva, Israel; c Department of Pediatrics, Soroka Medical Center, Israel

**Keywords:** Celiac disease, Celiac disease in adult population, Prevalence of celiac disease, Bedouin population

## Abstract

**Background:**

Celiac disease (CD) is a common, but often under-diagnosed condition with possible serious complications. CD, having a prevalence of about 1% is more common than once thought. Only limited research is available comparing differences between adults and children. A comprehensive Medline search was conducted. No data was found concerning the prevalence of CD among the adult Bedouin population.

**Methods:**

The research is retrospective and descriptive. The objective of our research was to determine the prevalence of the CD within adult and child Bedouin populations in urban Israel. A report of all of diagnosed CD patients extracted from the medical computerized information system (“Clicks”).

**Results:**

In our sample we found the prevalence was 0.51% in children and 0.12% in adults.

**Conclusion:**

In our opinion, one of reasons for the low prevalence level in the Bedouin community might be that typical CD symptoms are less prominent in Bedouin communities than in other communities. But no doubt hypo-diagnosis does exist. We suppose more advanced research about the nature and typical clinical manifestations of CD within the Bedouin population need to be investigated. Medical personnel working within the Bedouin community needs information concerning CD and the characteristics of diagnosis and treatment in the Bedouin community. The Bedouin community itself needs more information concerning CD and the importance of treatment, which could also improve early diagnosis and compliance.

## Introduction

Celiac disease (CD) is a common, but often under diagnosed condition with possible serious complications. CD is more common than once thought with a prevalence of about 1% [1-3]. CD is a common disorder in children and adults, but limited research is available comparing differences between various populations.

A comprehensive Medline search was conducted and no data was found concerning the prevalence of CD among the adult Bedouin population. There are only few known research investigations regarding CD in Arabic populations, and those were primarily interested in children [3-9].

CD has a strong genetic association with human leukocyte antigens (HLA). The HLA high-risk genotypes associated with CD are similar in Bedouin families and in Northern and Southern Europeans [4]. Undiagnosed CD is prevalent in infertile Arabic women, as well as in Arab women in general [6].

The IgA endomysium antibodies (EMA-IgA) and tissue transglutaminase antibodies (TTG-IgA) tests are both highly sensitive and highly specific, with values for both parameters exceeding 96% in most studies. No identifiable differences between adults and children are noted in response to these tests. The gold standard of diagnosis remains duodenal biopsy. A strict lifelong gluten-free diet is the cornerstone of management which improves symptoms and decreases complications of the disease.

Lately CD has become more frequently diagnosed due to the recognition of the atypical presentations [1].

The objectives of our research were: 1) Determining the prevalence of the CD among the Bedouin adult and child urban population in Israel; 2) Identifying the causes for the physicians’ reasons for suspecting CD. What complaint, symptom or laboratory findings cause the doctor to begin tests for CD, such as abdominal pain, elevated liver enzymes, iron deficiency anemia that does not respond to treatment etc; 3) Identifying diseases associated to CD, and comparison to the known data given in the literature.

## Research Methods and Structure

The research is retrospective and descriptive.

### Study population

In the research was included an urban Bedouin population which receives medical care service in the Rahat medical clinic, “Clalit health services” in largest Bedouin town in Israel.

### Inclusion criteria

Patients with diagnosis of CD were confirmed by positive result of a biopsy.

### Method for gathering information

A report of all of diagnosed CD patients extracted from the medical computerized information system (“Clicks”). Gathering information was done with a standard form (Appendix 1, www.gastrores.org).

### Statistical analysis

Continuous variables are shown as means. Categorical variables are described as frequencies. T-test and Chi-square tests were used to analyze statistically significant differences of continuous and categorical variables, respectively. Two-tailed p values less than 0.05 were considered statistically significant.

## Results

Family physician and pediatrician practices were analyzed. These services provide primary care services for 12619 adult/children- patients and pediatrician services are provided to10582 children (Table 1).

**Table 1:** Distribution by Age* and Gender

	Male		Female	
Children*	51.32%	N-5431	48.68%	N-5151
Adults*	47.47%	N-6001	52.53%	N-6618

*Child: age of diagnosis, up to 15 years; Adult: age of diagnosis, above 15 years.

The population was divided into two groups based on the age of diagnosis: those who were diagnosed before the age of 15 years (childhood celiac disease) and those diagnosed at the age of 15 or older (adult celiac disease).

According to the primary investigation 15 adult patients and 54 children were identified for inclusion.

The prevalence of childhood CD in our sample is 0.51 and of adult CD is 0.12 (Table 2).

**Table 2:** Prevalence According to Age of Diagnosis and Gender

	Male		Female		Total
Childhood Celiac Disease*	N = 22	40.74%	N = 32	59.26%	0.51%
Adult Celiac Disease	N = 3	20%	N = 12	80%	0.12%

Anemia is a main preliminary reason for examination (about half of all cases) (Table 3)

**Table 3:** Reasons for Investigation/Diagnosis

	Childhood celiac (N = 54)		Adult celiac (N = 15)		P Value
	N	(%)	N	(%)	
Anemia	27	(50)	7	(47)	1.000
Abdominal pain	11	(20)	0	(0)	0.105
Diarrhea	8	(15)	0	(0)	0.186
Family history	5	(9)	0	(0)	0.346
FTT	9	(17)			
Short stature	4	(7)			
Type I DM	1	(2)	0	(0)	1.000
Osteoporosis			1	(7)	
Elevated liver enzymes			1	(7)	
Others			6	(40)	

More than 90% of serologic tests were positive unequivocally among children while only about 50% the serologic tests of adults were positive (Table 4).

**Table 4:** Serologic Tests

		Childhood celiac (N = 54)		Adult celiac (N = 15)		P Value
		N	(%)	N	(%)	
EMA-IgA	Strongly positive	27	(61)	4	(31)	0.001
	Positive	15	(34)	3	(23)	
	Weakly positive	2	(5)	6	(46)	
TTG-IgA	Strongly positive	2	(20)	2	(33)	0.182
	Positive	7	(70)	1	(17)	
	Weakly positive	1	(10)	3	(50)	
Any serology	Strongly positive	27	(60)	4	(31)	0.001
	Positive	16	(36)	3	(23)	
	Weakly positive	2	(4)	6	(46)	

EMA-IgA: Anti-Endomysial Antibody IgA; TTG-IgA: Tissue Transglutaminase IgA Antibody.

The associated disease among our adult patients was primarily Iron Deficiency Anemia while among the children, Iron Deficiency Anemia, Failure To Thrive, and IBS were also present (Table 5, 6).

**Table 5:** Prevalence of Celiac Disease in Associated Autoimmune Conditions and Other Groups Where Case-Finding Should be Consider

Associated conditions	Prevalence of celiac disease (%)
Dermatitis herpetiformis	∼70
Type 1 diabetic patients	2 - 8
Thyroid disease	2 - 6
Addison's disease	1 - 12
Alopecia areata	1 - 2
Primary biliary cirrhosis	2 - 7
Autoimmune hepatitis	3 - 5
Idiopathic ataxia	1 - 7
Subfertility	4 - 8
Downs' syndrome	4 - 17
Iron-deficiency anaemia	3 - 7
Irritable bowel symptomatology	0 - 11
Peripheral neuropathy	Up to 23

(British Medical Bulletin Advance Access originally published online on December 10, 2008 ); British Medical Bulletin 2008 88 (1):157-170; doi:10.1093/bmb/ldn044.

**Table 6:** Background Diseases

	Childhood celiac (N = 54)		Adult celiac (N = 15)		P Value
	N	(%)	N	(%)	
Dermatitis herpetiformis	0	(0)	0	(0)	1.000
Type I DM	1	(2)	0	(0)	1.000
Thyroid disease	1	(2)	1	(7)	0.390
Addison's disease	0	(0)	0	(0)	1.000
Alopecia areata	0	(0)	0	(0)	1.000
Primary biliary cirrhosis	0	(0)	0	(0)	1.000
Autoimmune hepatitis	0	(0)	0	(0)	1.000
Idiopathic ataxia	0	(0)	1	(7)	0.217
Subfertility	0	(0)	0	(0)	1.000
Down's syndrome	0	(0)	0	(0)	1.000
Iron deficiency anemia	33	(61)	8	(53)	0.767
IBS	20	(37)	0	(0)	0.008
Peripheral neuropathy	0	(0)	0	(0)	1.000
FTT	14	(31)			
Short stature	7	(16)			

DM: Diabetes Mellitus; IBS: Irritable bowel symptoms; FTT: Failure to Thrive.

## Discussion

As mentioned before, the prevalence of CD is about 1% in the general population, according to the most of the research found. In our population we found the prevalence as 0.51% in children and 0.12% in adults. No doubt hypo-diagnosis does exist. In our opinion, one of reasons for the low prevalence level in the Bedouin community could be that CD symptoms are less prominent in Bedouin communities than in other communities. As a result people come to doctor with complaints atypical for CD. Support for this hypothesis is indicated in Table 7 were the average age of diagnosis in children is 12, compared to average age of 1-2 years shown in other medical data [10]. Neither autoimmune disease nor malignancy was confirmed in any of our patients. Until recently, it was reported that one of main reason for the mortality from CD is significant weight loss as a sign for the refractive form of CD [11]. No patients in our sample suffered from prominent weight loss, despite not keeping a gluten free diet by the majority of our patients and we did not notice significant worsening of the current disease.

**Table 7:** Other Relevant Parameters at the Time of Diagnosis

	Childhood celiac (N = 54)		Adult celiac (N = 15)		P Value
	Mean	(SD)	Mean	(SD)	
Age of Diagnosis	12	(4)	35	(9)	< 0.001
Female Gender	32	(59)	12	(80)	0.225
BMI	16.3	(3.0)	22.6	(4.5)	< 0.001
Hb	10.7	(1.8)	11.2	(1.9)	0.361
Iron	31	(24)	34	(28)	0.746
ELFT1/ALT	39	(27)	31	(14)	0.261
ELFT2/AST	24	(16)	28	(21)	0.500

ELFT1: Elevated Liver Functional Test; ALT: Alanin Aminotransferase; ELFT2: Elevated Liver Functional Test; AST: Aspartate Aminotransferase.

How can early diagnosis be achieved? For adults Iron Deficiency Anemia which does not respond to treatment with supplemental iron might be a sign for obligatory testing for CD (at least for a serologic test to start with). In children there are three signs: low BMI, FTT and Iron Deficiency Anemia for considering tests to exclude presence of CD.

One of the reasons perplexing the diagnosis was poor compliance of some patients. For example, four adult patients having positive serology results for CD refused to have a biopsy. Therefore the diagnosis of CD had not been confirmed. Attempts to screen relatives of CD patients partially failed as well. We know that a delay in the diagnosis of CD may prolong morbidity and increase mortality. The improvement of awareness of population about CD in different ways (personal doctor-patient relationships, lectures, increasing awareness of research results, etc.) will improve compliance of patients and will increase early diagnosis of CD.

### Conclusion

The results of our research indicate some hypo-diagnosis of CD among the Bedouin population in southern Israel. Further research about the nature and typical clinical manifestations of CD among the Bedouin population is required so that medical personnel will be alerted to the signs for testing, allowing for early diagnosis. No doubt there is a necessity to increase awareness and understanding concerning CD within the Bedouin community itself through a program providing information and improving patient education, early diagnosis of CD, and most important, compliance with treatment for the condition.
